# Monitoring Population Exposure to Low‐ and No‐Calorie Sweeteners via Pooled Urine Analysis

**DOI:** 10.1002/fsn3.71795

**Published:** 2026-04-21

**Authors:** Nicole S. Schröter, Jake W. O'Brien, Richard Bade, Leisa‐Maree L. Toms, Peter Hobson, Daman Langguth, Jochen F. Mueller

**Affiliations:** ^1^ Queensland Alliance for Environmental Health Sciences (QAEHS) The University of Queensland Woolloongabba Queensland Australia; ^2^ School of Public Health and Social Work Queensland University of Technology Kelvin Grove Queensland Australia; ^3^ Sullivan Nicolaides Pathology Bowen Hills Queensland Australia; ^4^ Minderoo Centre—Plastics and Human Health Woolloongabba Queensland Australia

**Keywords:** consumption trends, human biomonitoring, low‐ and no‐calorie sweeteners, pooled urine, population exposure, young children

## Abstract

Low‐ and no‐calorie sweeteners (LNCS) are increasingly used as sugar substitutes in processed foods and sweetened beverages to reduce energy intake while maintaining taste. Given that poor diet remains a major contributor to non‐communicable diseases, LNCS offer a strategy to lower caloric intake. However, ongoing concerns about their health effects highlight the need for a clearer understanding of LNCS exposure patterns within vulnerable groups of the population. Data on LNCS exposure and consumption trends, especially in the younger population, remain limited. Pooled urine samples were analyzed for four LNCS (acesulfame, sucralose, cyclamate, and saccharin) using Liquid Chromatography Tandem Mass Spectrometry (LC–MS/MS). Urinary concentrations were determined for various demographics (sex, age, and remoteness) to identify patterns of exposure, with focus on the younger population (< 5 years). Measured concentrations across all pools (*n* = 135) containing 3375 individuals ranged from 0.1 to 40 mg/L for acesulfame, 0.04–30 mg/L for saccharin, 0.01–30 mg/L for cyclamate, and 0.01–1.3 mg/L for sucralose. Saccharin showed significantly higher concentrations among children and adolescents (0–15 years) compared to adults (15–60 years). No significant differences were observed between sexes or age groups for acesulfame, cyclamate, and sucralose. LNCS concentrations did not vary significantly by remoteness. Esti‐mated average daily saccharin intake was 600 μg/kgbw/day for children aged 0–5 years, representing 12% of the acceptable daily intake (ADI), compared to 25 μg/kgbw/day (0.5% of ADI) in adults. In the case of saccharin, the highest concentrations were observed in young children, which may represent a higher risk of potential adverse health outcomes. Average urinary concentrations of all LNCS were determined, serving as indicators of average population intake.

## Graphical abstract

The graphical abstract illustrates trends in low‐ and no‐calorie sweeteners exposure across the Australian population. It highlights differences between males and females across six age groups, as well as variation across five remoteness categories. An example biomarker, saccharin, is shown with concentrations plotted by age group, demonstrating how exposure levels vary across demographic strata.

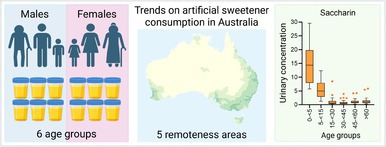

## 1 Introduction

Sweet taste stimulates the brain's reward system by triggering dopamine release, a pathway also involved in responses to social interaction, sexual stimuli, and addictive substances. Consequently, highly processed foods with high levels of sweeteners have the potential to induce addictive‐like responses in certain individuals, which may result in overconsumption and adverse health effects (Murray et al. 2016). As the tongue detects sweetness, the urge to consume it is driven by gut signals (Liu and Bohórquez 2022). Low‐ and no‐calorie sweeteners (LNCS) can provide this sweetness without calories. Regulatory approval of LNCS also varies by country and compound. For instance, cyclamate is banned in the United States and South Korea due to toxicity concerns, while it is permitted in other regions (Oser et al. 1976; OFR/GPO et al. 1977). Others, like saccharin, are widely authorized under specific conditions. In Australia, approved artificial sweeteners are saccharin, sodium cyclamate (cyclamate), acesulfame potassium (acesulfame), sucralose, aspartame and its modified forms, advantame and neotame, besides other natural low and no‐nutritive sweeteners such as Stevia (Food Standards Australia New Zealand 2023). Of these, saccharin, acesulfame and sucralose are excreted unchanged in urine after passing undigested through the gastrointestinal tract, but the minimal absorption of sucralose leads to lower excretion in urine than for saccharin and acesulfame (Renwick 1986; Magnuson et al. 2016). Cyclamate gets only partially absorbed and excreted in urine; the rest can be converted into cyclohexylamine depending on the individual's ability to do so (Renwick and Williams 1972). Further details can be found in Table S1. Saccharin, cyclamate, acesulfame and sucralose were selected for this study as the LNCS most prominently used in sweetened foods and beverages worldwide. Aspartame was excluded because it cannot be measured in urine due to its rapid and complete absorption and metabolism. With a focus on artificial sweeteners, natural low‐ or no‐calorie sweeteners were also excluded from the study. Maximum permitted concentrations of the selected LNCS in Australian processed food products can be found in Table 1.

**TABLE 1:** Maximum permitted concentrations for sweeteners as food additives in different processed and packaged food products in Australia.

Sweetener	Common maximum levels (mg/kg)	Highest levels (mg/kg)
Acesulfame	500 (dairy, juice, coffee, sauces, jelly), 1000–2500 (ice cream, brewed soft drinks), 3000 (flavored drinks, fruit spreads)	5000 (chewing gum)
Saccharin	50–160 (soft drinks, jelly), 150 (flavored drinks), 200 (medical food)	1500 (sauces, fruit spreads)
Cyclamate	350–1350 (flavored drinks, spreads, sauces), 1600 (jelly)	20,000 (chewing gum)
Sucralose	250 (brewed soft drinks)	2500 (confectionery)

*Source:* Food Standards Australia New Zealand (2024).

LNCS consumption has been estimated in various populations using dietary surveys, food diaries, and database records (Bär and Biermann 1992; Leclercq et al. 1999; Wilson et al. 1999; Ilbäck et al. 2003; Martyn et al. 2016; Sylvetsky, Jin, et al. 2017; Garavaglia et al. 2018). However, these studies often depend on self‐reported data, which is susceptible to recall bias and under‐reporting. Moreover, variations in food labelling and database completeness can further compromise their accuracy. Existing urinary concentration data are primarily derived from intake‐excretion and dose‐response studies conducted in the adult population, with very limited sample sizes (Wilson et al. 1999; Logue et al. 2017; Sylvetsky, Walter, et al. 2017; Weinborn et al. 2021). Many focus on individual compounds and are conducted under controlled study conditions, which may not reflect real‐world consumption patterns. Moreover, no urinary concentration data for LNCS in young children (0–< 5 years) were identified in the existing literature (Baker‐Smith et al. 2019).

To address these limitations, pooled urine analysis provides a biomarker‐based approach for objectively measuring LNCS concentrations, offering a more reliable reflection of consumption patterns and enabling a socio‐demographic exposure assessment at the population level. Pooled urine analysis is an established approach that has been used in Australia since 2012 to assess age, sex and regional differences in chemical concentrations, and to investigate temporal trends over more than a decade, including phthalates (Gomez Ramos et al. 2016; Tang et al. 2020), polycyclic aromatic hydrocarbons (Thai et al. 2020, 2016), pesticides (Heffernan et al. 2016), bisphenol A (Heffernan et al. 2013), personal care products chemicals (Heffernan et al. 2015), benzotriazoles and benzothiazoles (Que et al. 2024) and organophosphate flame retardant (Van den Eede et al. 2015). In addition to being cost‐effective and time‐efficient, pooled urine analysis allows for the inclusion of vulnerable or hard‐to‐reach subpopulations, such as young children.

The aims of this study are to: (1) establish average concentrations of LNCS in pooled urine samples, stratified by age, sex, and remoteness; (2) examine potential trends in LNCS consumption patterns by these parameters; and (3) extrapolate the concentrations to estimate the total annual LNCS consumption across the Australian population for a possible global comparison.

## 2 Materials and Methods

### 2.1 Pooling Strategy

The samples used to create the pools were obtained from surplus pathology urine samples collected during routine clinical screenings of the general population in late 2019 and early 2020 by Sullivan Nicolaides Pathology (SNP) in Bowen Hills, Queensland, Australia. The individual samples originated from various regions across Australia (2019/20), with the following distribution: Queensland 80% (1488 female and 1221 male samples), Northern Territory 12% (264 female and 147 male samples), New South Wales and the Australian Capital Territory 7% (118 female and 126 male samples) and the remaining states 1% (5 female and 6 male samples). They were subsequently de‐identified and pooled to ensure anonymity; only sex, date of birth, postcode, and collection date were recorded. All samples were pooled based on six age groups (young children = 0–< 5, adolescents = 5–< 15, young adults = 15–< 30, adults = 30–< 45 and 45–< 60, and seniors = ≥ 60 years old), sex (female and male) and five remoteness classes (Major Cities, Inner Regional, Outer Regional, Remote, and Very Remote). The remoteness stratification of the samples was based on the Australian Statistical Geography Standard (ASGS) used by the Australian Bureau of Statistics (ABS), which uses the Accessibility/Remoteness Index of Australia Plus (ARIA+), developed by the University of Adelaide. ARIA scores reflect road distances from populated localities to service centres (Australian Bureau of Statistics 2021; The Australian Centre for Housing Research | University of Adelaide 2023). The creation of each independent pool for the remoteness, age, and sex stratification was achieved by combining 800 μL of each of the 3375 individual urine samples into 135 distinct pools, resulting in 25 individuals/pool and a final volume of 20 mL for each pool. The distribution of these pools is documented in the Table S2.

### 2.2 Sample Preparation

The urine pools are stored at a temperature of *−*20°C within glass scintillation vials. The pools were thawed, aliquots of 500 μL of each pool were taken, centrifuged twice at 16,350*g* for 10 min and the supernatant was recovered. Using 10 μL urine, each pool was diluted 100× with an aqueous solution made of 99% purified water (MilliQ system, 0.22‐μm‐filtered, 18.2 MΩ/cm; Millipore), 1% methanol (Merck Pty Ltd) and 0.1% acetic acid (Sigma). Acesulfame‐d^3^ (TRC Inc.) was used as an internal standard (IS) for acesulfame and as a surrogate IS for saccharin, cyclamate and sucralose; 10 ng (in 10 μL methanol) of the IS was added to each sample, adding up to a final volume of 1000 μL. A 10‐point calibration curve ranging from 0.1 to 2000 μg/L was prepared with the aqueous solution. Blanks (BLK), spiked samples (SPK), and duplicate samples (DUP) were prepared with 10 ng/mL IS and native analytes using the same protocol. A total of 10% of the samples were randomly selected and prepared as either DUP or SPK, resulting in 14 pools (7 DUP, 7 SPK).

### 2.3 Liquid Chromatography Tandem Mass Spectrometry (LC–MS/MS) Analysis

The four selected LNCS were simultaneously determined in pooled urine using an established method, with only key analytical details summarized here; comprehensive methodological information is available in the original source (Li et al. 2020). Quantification was performed using a Sciex 6500+ QTRAP mass spectrometer (Sciex, Ontario, Canada) coupled to a Shimadzu Nexera 2 HPLC system (Shimadzu Corp., Kyoto, Japan), equipped with an ESI source operating in negative ionization and Multiple‐Reaction Monitoring (MRM) mode. Chromatographic separation was achieved using a Luna Omega Polar C18 column (100 Å, 1.6 μm, 50 × 2.1 mm) with a Kinetex EVO C18 pre‐column (5 μm, 30 × 2.1 mm), an oven temperature at 45°C and a flow rate of 0.4 mL/min with a gradient starting at 0% B held for 1.5 min than ramped to 20% B for 2.0 min, ramped to 99% B for 1.5 min, held at 95% for 1.9 min and followed a 0% B isocratic hold for 3.0 min (B = 95% methanol, 5% Milli‐Q and 0.1% acetic acid). Elution was directed to an IonDrive Turbo V source operating in ESI mode at 480°C. The source parameters were set as follows: curtain gas at 30, ion source gas 1 and gas 2 at 70. The ionspray voltage was −4500 V, and the entrance potential was set to −10 V.

Quantification of biomarkers was performed through IS and isotope dilution with LC‐MS/MS. The standards, methanol (Merck Pty Ltd) and acetic acid (Sigma) used for the analysis were all of analytical grade. Water purification was achieved using a Milli‐Q system (0.22‐μm‐filtered, 18.3 MΩ/cm; Millipore). The injection volume for each sample was 5 μL for quantifying acesulfame, saccharin and cyclamate and 10 μL for sucralose.

### 2.4 Quality Assurance and Quality Control

A BLK and a continuing calibration verification (CCV) standard were injected in a typical batch after every 10 samples. The resulting data was quantified using the *MultiQuant* software (SCIEX, Toronto, Canada). Most of the peaks had a minimum of 6 points across the full width at half height. The linearity of the calibration curve was achieved between 0.2 and 2000 μg/L (*n*=9) for acesulfame, saccharin and sucralose and 2–2000 μg/L (*n* = 6) for cyclamate, and the correlation coefficient was *R* ^2^ > 0.99. This being a dilute and shoot method without any extraction steps, the calculation for the recovery of the IS and native standards in samples compared to the non‐extracted side spike (NESS) will be defined as direct recovery.

### 2.5 Intake Estimation

The average daily intake was calculated using mean urinary volumes (*V*_urine_) for young children (0–5 years) (Beckford et al. 2019) and individuals older than 5 years (González‐Mariño et al. 2017). The excretion factor (EF) represents the fraction of each compound excreted in urine within 24 h of ingestion. Equation (1) was used to calculate the average daily intake (DI_calculated_) derived from the mean urinary concentration of the urine pools for each age group (*c*_measured_). Equation (2) utilized this value to extrapolate the annual intake in the population (Intake_population_) in kilograms per year (kg/year) for each LNCS per age group. The population distribution for each age group was used to calculate the total consumption for the Australian population (*N*), which was estimated to be 25.5 million in 2019 (2019 data was used to match urine samples collection year) (Australian Bureau of Statistics 2019). Comprehensive parameter values and detailed data can be found in the Table S8.

(1) $${\mathrm{DI}}_{\text{calculated}}=\frac{c_{\text{measured}}\times V_{\text{urine}}}{\mathrm{EF}}$$

(2) $${\text{Intake}}_{\text{population}}={\mathrm{DI}}_{\text{calculated}}\times \frac{N\times 365}{10^{6}}$$

### 2.6 Statistical Analysis

The Shapiro‐Wilk test was applied to assess the normality of the data (*α* = 0.05). Most results did not follow a normal distribution (*p* < *α*). Therefore, Kruskal‐Wallis and Dunn's post hoc tests were performed to identify significant differences among the multiple remoteness factors and age groups. The Mann‐Whitney test was performed for the sex comparison (GraphPad Prism, Version 9.5.1.733, GraphPad Software LLC). A value of *p* < 0.05 was considered statistically significant. Outliers were identified using the Robust Regression and Outlier Removal (ROUT) method with *Q* = 1%, a statistical approach designed to detect outliers in datasets with non‐normal distributions. This method leverages the False Discovery Rate (FDR), a critical parameter in non‐linear regression, to systematically identify data points that deviate significantly from the expected distribution. The ROUT method constructs a model based on the dataset, applying the FDR threshold to distinguish and exclude outliers while preserving the integrity of the analysis. The results are presented as mean with range unless otherwise specified.

## 3 Results

### 3.1 Urine Pools

**FIGURE 1:**
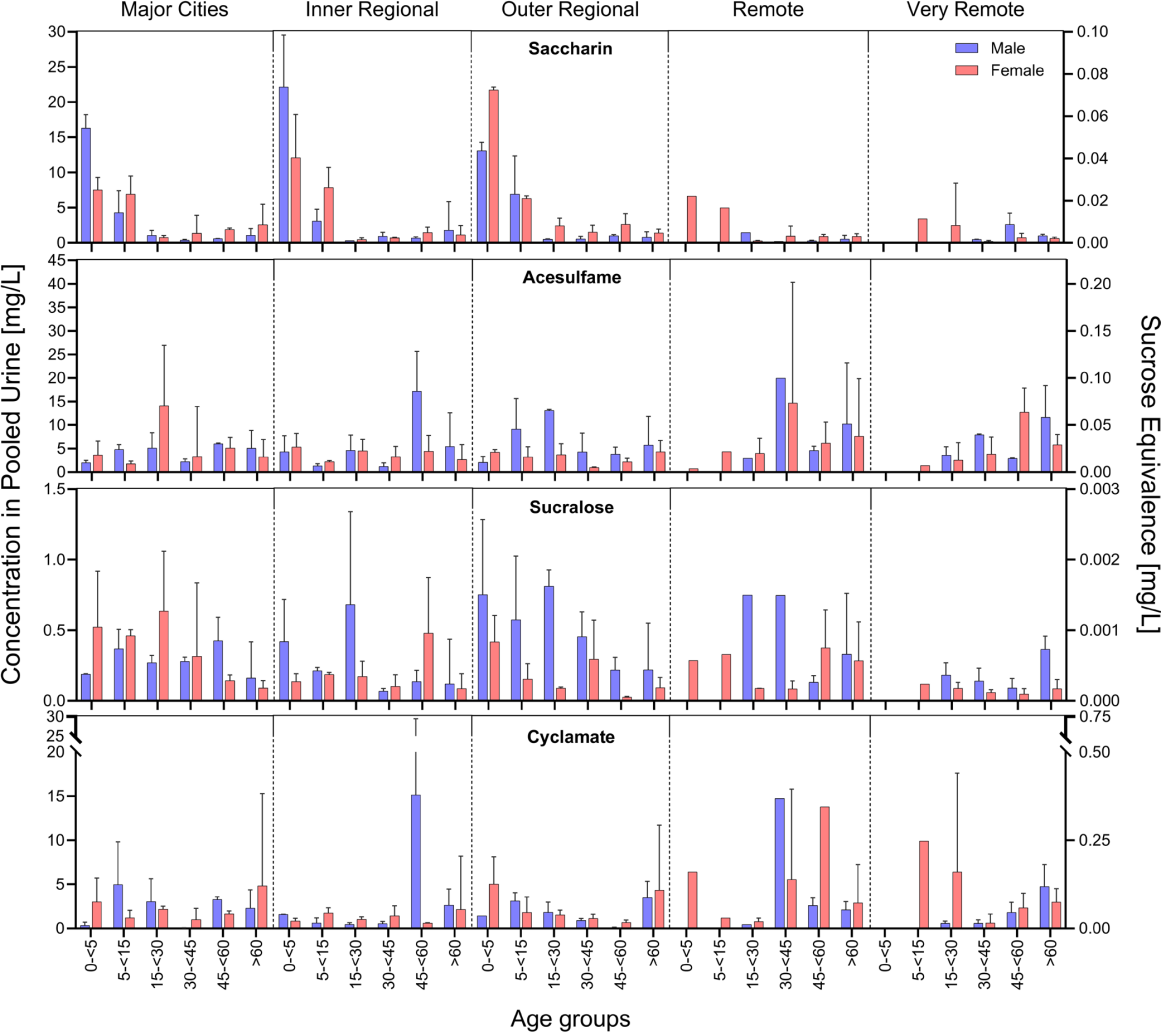
Concentrations of sweeteners in pooled urine (left axis) and corresponding sucrose‐equivalent concentrations (right axis), stratified by age, sex, and remoteness factor; each pool contained 25 individuals from the same age group, sex, and remoteness factor; displayed are the means (bars) with the upper ranges (error bars) for each category; blue = male, red = female; each window from the left to the right displays the different remoteness factors (top) for all age groups of that window (*x*‐axis); no bar = no pools, only bar = one pool, bar with error bar = two or more pools.

Of the 135 pooled urine samples, detection frequencies were 100% for acesulfame and saccharin, and 98.5% for sucralose and cyclamate. Concentrations ranged from 0.1–40 mg/L (acesulfame), 0.04–30 mg/L (saccharin), 0.01–30 mg/L (cycla‐mate), and 0.01–1.3 mg/L (sucralose) (Figure 1). The established mean urinary concentrations for all age groups and sweeteners can be found in (Table 2).

**TABLE 2:** Established mean urinary concentrations (mg/L) of sweeteners in pooled urine stratified by age groups.

Age groups (years)	0–< 5	5–< 15	15–< 30	30–< 45	45–< 60	≥ 60
Artificial sweetener	Mean urinary concentrations (mg/L)					
Saccharin	15	5.7	1.2	0.9	1.2	1.3
Acesulfame	3.4	3.6	5.5	5.4	6.8	5.7
Cyclamate	2.5	2.7	2.5	2.0	3.6	3.1
Sucralose	0.4	0.3	0.3	0.2	0.2	0.2

### 3.2 Method Validation

IS direct recovery in the calibration curve, the BLKs (*n* = 11), and CCVs (*n* = 19) was between 81% and 110%, and in the urine samples (*n* = 87) between 62% and 93%. Native standard direct recovery in the calibration curves and CCVs was between 84% and 120% for all LNCS. Native standard direct recovery in spiked urine samples compared to the NESS sample was between 83% and 140% for acesulfame, cyclamate, and saccharin (with one saccharin outlier at 180%) and between 110% and 150% for sucralose. The percentage difference of the DUP samples (*n* = 13) ranged from 0.1% to 6.6% for acesul‐fame, cyclamate, and saccharin and by 0.3%–19% for sucralose. A The Lower Limit of Quantification (LLOQ) study was conducted according to the ICH guidelines (European Medicines Agency (EMA) and Committee for Medicinal Products for Human Use 2022). The LLOQ was determined using the mean and standard deviation (SD) of repeated injections (*n* = 3) of replicate samples (*n* = 7) at the lowest calibration point for each LNCS. The LLOQ in aqueous solution was found to be 0.2, 0.3, 0.3, and 2.3 μg/L for acesulfame, saccharin, sucralose, and cyclamate, respectively. The LLOQ in 100‐fold diluted synthetic urine was determined to be 0.3, 0.3, 0.3, and 2.9 μg/L for acesulfame, saccharin, sucralose, and cyclamate, respectively.

### 3.3 Statistical Outcomes

#### 3.3.1 Sex

The Mann‐Whitney test was used to assess differences in LNCS concentrations between sexes, independent of age group or remoteness. Statistically significant differences (*p <* 0.05) were observed for acesulfame and sucralose, with higher average concentrations in males (6.0 ± 5.5 and 0.3 ± 0.3 mg/L, respectively) than in females (4.8 ± 6.2 and 0.2 ± 0.2 mg/L). Full statistical outputs are available in Table S3. The mean values being greater than the medians, along with large standard deviations, suggest a positively skewed distribution. This was confirmed using the adjusted Fisher–Pearson coefficient of skewness (g), where *g >* 1 for both sexes across all four LNCS, indicating high positive skewness in the data.

#### 3.3.2 Age

**FIGURE 2:**
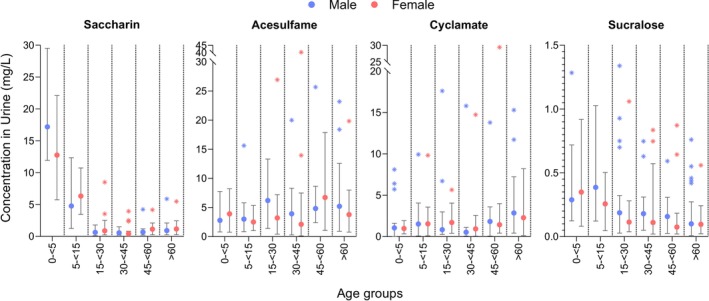
Trends of sweeteners in pooled urine stratified by age and sex; numbers of pools for the different age groups and sex were *n*_male (0–< 5)_ = 6, *n*_female (0–< 5)_ = 7, *n*_male (5–< 15)_ = 6, *n*_female (5–< 15)_ = 8, *n*_male (15–< 30)_ = 9, *n*_female (15–< 30)_ = 15, *n*_male (30–< 45)_ = 9, *n*_female (30–< 45)_ = 16, *n*_male (45–< 60)_ = 10, *n*_female (45–< 60)_ = 9, *n*_male (≥ 60)_ = 20, *n*_female (≥ 60)_ = 20; each pool consisted of 25 individuals. Data are presented as means (dots) with ranges (error bars) and outliers represented as stars (*), calculated using the ROUT method (*Q* = 1%).

Statistically significant differences (*p <* 0.05) across age groups (irrespective of sex and remoteness) were observed for saccharin, sucralose and cyclamate concentrations using a Kruskal‐Wallis test. Saccharin concentrations were highest in young children (0–< 5 years, 15 mg/L) and adolescents (5–< 15 years, 5.7 mg/L) compared to adults (15–> 60 years, 0.9–1.3 mg/L). Sucralose concentrations were higher in young children (0–< 5 years, 0.4 mg/L) than in seniors (≥ 60 years, 0.2 mg/L), while cyclamate concentrations were lower in adults (30–< 45 years, 2.0 mg/L) than in seniors (≥ 60 years, 3.1 mg/L) (Figure 2). The test was repeated for age and sex, regardless of remoteness, to assess combined effects, whereby sucralose showed statistical significance in females overall, no differences between the distinct age groups were found. Further details for both analyses are available in Tables S4 and S5.

#### 3.3.3 Remoteness

Significant variation across five remoteness categories (independent of age and sex) was found for saccharin and sucralose (*p <* 0.05), but not for acesulfame or cyclamate using a Kruskal–Wallis test. Given the strong age‐related influence on the urinary concentration of saccharin, interpretation without age stratification can lead to skewed conclusions. Thus, no further analysis for saccharin was performed based on the remoteness factor. Post hoc analysis identified a significant difference in sucralose concentrations between “Major Cities” (0.3 mg/L, *n* = 33) and “Very Remote” areas (0.1 mg/L, *n* = 23). It is important to mention that no urine pools were available for males < 15 years and females < 5 years in remote and very remote areas, see Figure 1. Full results and visualizations are provided in Tables S6, S7 and Figure S1.

### 3.4 Intake Estimation

Urinary LNCS concentrations were used to estimate daily intake across all age groups. Equation (1) calculated the mean daily intake DI_calculated_ for each LNCS using age‐specific urinary volumes and compound‐specific excretion factors (Table 3).

**TABLE 3:** Estimated average daily intake (mg/day) per person of sweeteners for each age group.

Age groups (years)	0–< 5	5–< 15	15–< 30	30–< 45	45–< 60	*≥* 60
Sweetener	DI_calculated_ (mg/day)					
Saccharin	8.5	9.4	2.1	1.5	2.1	2.2
Acesulfame	1.8	5.7	8.7	8.6	11	9.0
Cyclamate	3.3	11	9.9	7.7	14	12
Sucralose	1.6	3.8	3.9	2.8	2.4	2.0

When calculating the mean daily intake (mg/kgbw/day) value for young children aged 0–< 5 years, adjustments were made for the lower urinary volume and weight using saccharin as an example. An average body weight of 14 kg (World Health Organisation and Department of Nutrition for Health and Development 2009), representative of a 3‐year‐old, the predominant age contributing to the urine pools, was applied. Based on a daily urinary volume of 0.53 L/day (Beckford et al. 2019), an excretion factor of 0.92 (Byard et al. 1974), and the mean urinary saccharin concentration of 15 mg/L, the estimated daily intake was 0.6 mg/kgbw/day.

**FIGURE 3:**
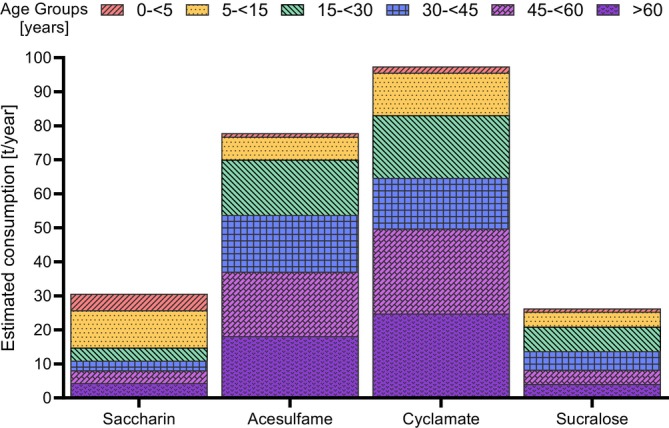
Population adjusted estimated total annual consumption of each sweetener by age group in 2019 in Australia; age groups are shown in years; each color represents the estimated proportion of the total tonnes consumed annually by each age group.

Equation (2) was used to estimate the annual intake of each LNCS across the Australian population by age group (Figure 3), yielding a total consumption of 230 t/year for all four LNCS: 31 t/year (saccharin), 26 t/year (sucralose), 78 t/year (acesulfame), and 97 t/year (cyclamate). For saccharin, the highest concentrations were observed in young children, with individuals under 15 years accounting for an estimated 52% of total consumption. In contrast, more than 80% of acesulfame, cyclamate, and sucralose consumption was attributed to individuals aged 15 years or older. Further detailed information can be found in Table S8.

## 4 Discussion

This study is the first to systematically analyze pooled urine samples from a broad age range, including a significant proportion of children under 15, to assess exposure to low‐ and no‐calorie sweeteners (LNCS). By incorporating age‐ and sex‐stratified sampling, average urinary concentrations for four widely used LNCS (saccharin, acesulfame, sucralose, and cyclamate) across six age groups were established (Table 2). These findings provide a crucial baseline for future monitoring and exposure assessments, particularly given the scarcity of age‐specific exposure data in the existing literature. The study revealed that age was the most significant factor influencing urinary saccharin concentrations, while remoteness and sex had a negligible impact on these concentrations.

Children and adolescents are especially important to study, as their dietary choices are heavily shaped by parental habits and preferences, cultural influences, and aggressive marketing strategies by the food industry (Kearney 2010; Nestle 2019; Saygi and Shipman 2021; Boyland et al. 2022). These factors contribute to higher consumption of artificially sweetened foods and beverages in younger age groups, raising concerns about early and sustained exposure to LNCS. Therefore, establishing population‐level reference values for urinary LNCS concentrations in children and adolescents, who are often underrepresented in exposure research, is vital for the formulation of public health policies and dietary guidelines.

Saccharin concentrations in adults ranged from 0.9 to 1.3 mg/L (means, *n* = 2700, > 15 years) in this study, which is higher than in previously reported individual‐level studies, such as the baseline concentration after fasting of 0.4 mg/L (mean, *n* = 23 females, 18–45 years) reported in the United States (Weinborn et al. 2021), and a concentration of 0.1 mg/L (median, *n* = 54, 22‐62 years) among adults in China (Zhang et al. 2016). Similarly, average sucralose concentrations in our cohort (0.3 mg/L, *n* = 550, 30–< 45 years) were higher than earlier findings (mean, 0.1 mg/L, *n* = 54, 22–62 years) from the United States (Sylvetsky, Walter, et al. 2017). These discrepancies are likely due to differences in sample size and population diet in the country of origin. Whereas pooling reduces individual variability, it improves the estimation of population‐level means (Caudill 2008; Heffernan et al. 2014).

LNCS are commonly present in beverages, bakery items, confectionery, oral hygiene products, and pharmaceuticals, including children's medicine (Food Stan‐dards Australia New Zealand 2023). For instance, Saccharin is commonly used in flavored drinks and confectionery, ranks second in sweetened cough syrups, and is the most prevalent sweetener in toothpastes and mouthwashes (Food Standards Australia New Zealand 2023; U.S. Food and Drug Administration 2014; Eccles 2020). Regional differences exist, with Oceania and European products typically containing a single LNCS in beverages and bakery products, while North American items often feature multiple LNCS (Billy Yin Sing et al. 2021). Given their ubiquity, LNCS are emerging as valuable biomarkers of sweetened processed food and beverage consumption.

### 4.1 Children

Intake estimation indicated that children aged 0–< 5 years had significantly higher exposure to saccharin (0.6 mg/kgbw/day, 12% of the ADI) compared to adults (0.02–0.03 mg/kgbw/day, 0.5% ADI), even after accounting for body weight and urinary volume (Table 3). This aligns with findings that children are disproportionately exposed to LNCS due to their smaller body size and higher consumption of sweetened beverages and medicines per unit of body weight (Martyn et al. 2016; Garavaglia et al. 2018; Martínez et al. 2020). The estimated intake of the other sweeteners for children aged 0–< 5 years was 0.9%, 2.1%, and 2.3% for acesulfame, cyclamate, and sucralose, respectively. All ADI values can be found in the Table S1.

LNCS, including saccharin, sucralose, and acesulfame, have also been detected in human milk, amniotic fluid, and cord blood, suggesting exposure begins in utero and continues through infancy (Cohen‐Addad et al. 1986; Sylvetsky et al. 2015; Halasa et al. 2020). While the evidence is inconclusive, it is plausible to hypothesize that these exposure pathways lead to increased systemic concentrations in infants.

Although saccharin is currently considered safe within the ADI, its elevated intake among children has previously raised regulatory concerns. In 1978, the European Commission advised against its use in infant food products (Official Journal of the European Communities 1978). However, recent changes, such as the European Food Safety Authority's (EFSA) 2024 decision to raise the ADI from 5 to 9 mg/kgbw/day due to an improved manufacturing process considered less genotoxic, contrast earlier cautionary approaches (Castle et al. 2024). This occurred previously in 1993, when the Joint FAO/WHO Expert Committee on Food Additives (JECFA) increased the acceptable daily intake (ADI) for saccharin from 2.5 to 5 mg/kgbw/day, based on a no‐observed‐effect level (NOEL) of 500 mg/kgbw/day observed in rat studies (World Health Organisation 1993). This historical evolution underscores the importance of adopting a precautionary approach, particularly concerning early‐life exposure.

### 4.2 Population‐Level Trends

Population‐corrected intake estimates indicate that 52% of total saccharin consump‐tion was attributable to individuals under 15 years of age, while more than 80% of acesulfame, cyclamate, and sucralose intake was accounted for by individuals aged 15 years and older (Figure 3). Total LNCS consumption in 2019 (230 t/year) exceeded previous estimates based on wastewater‐based epidemiology (WBE) data (139 t/year) (Li et al. 2020). Wastewater‐based estimates may underestimate actual consumption due to degradation, loss or transformation of sweeteners during sewage transport, as well as variations in population coverage. In contrast, market‐based estimates (1778 t/year) rely on sales data, which do not necessarily translate to actual intake, as they may include wastage, stockpiling, or export (Statista Market Insights 2024). Additionally, the market data encompasses a wider range of sweeteners beyond the four analyzed in this study, further contributing to the higher estimate. While the WBE and pooled urine approach each provide valuable insight, they reflect different aspects of LNCS consumption and should be interpreted as complementary data. Research on dietary patterns in Australia found, lower socioeconomic status is linked to less healthy food choices, including fewer fruits and vegetables and high‐fat. At the same time, higher‐income individuals and women tend to prioritize taste over price (Turrell et al. 2002, 2004; Ward et al. 2012). These demographic patterns show that broader structural factors such as food containing LNCS availability, accessibility and marketing, also influence LNCS consumption. The data present do not directly measure these effects, they provide important contextual information for the interpretation of national trends.

### 4.3 Limitations

A key limitation of our study is the use of surplus pathological urine samples from individuals undergoing specific clinical tests, such as for urinary tract infections, diabetes (glucose monitoring), renal function, sexually transmitted infections, and drug screening, may introduce selection bias. Furthermore, the absence of detailed participant information due to the pooling and de‐identification restricts the ability to explore associations with health status, lifestyle, dietary patterns, or socioeconomic status. Additionally, the over‐representation of samples from Queensland and the Northern Territory may limit the generalization of the findings to the broader Australian population. While pooled sampling enabled efficient estimation of group‐level exposure, it masked intra‐group variability.

## 5 Conclusion

This study is the first to provide population‐level urinary reference values for LNCS across a wide age range and the sexes in Australia, using the pooled urine analysis approach. The finding that children under 5 years have more than a 10‐fold higher intake (per kg body weight) of saccharin compared to adults highlights the need for future research into exposure trends and potential long‐term metabolic consequences of early‐life exposure. Despite these limitations, this study offers valuable baseline data and highlights the feasibility of pooled urine sampling as a practical, population‐level method for assessing real‐world exposure trends. Future studies should prioritize investigating the interactions between socioeconomic factors, other dietary biomarkers, and LNCS exposure in younger populations, using routine and longitudinal monitoring to capture temporal trends.

## Author Contributions

**Nicole S. Schröter:** conceptualization, data curation, formal analysis, investigation, methodology, validation, writing – original draft, writing – review and editing, visualization. **Jake W. O'Brien:** supervision, validation, writing – review and editing. **Richard Bade:** validation, writing – review and editing, supervision. **Leisa‐Maree L. Toms:** funding acquisition, writing – review and editing. **Peter Hobson:** project administration, resources. **Daman Langguth:** project administration, resources. **Jochen F. Mueller:** conceptualization, funding acquisition, project administration, supervision, writing – review and editing.

## Funding

The Queensland Alliance for Environmental Health Sciences (QAEHS) at The University of Queensland (UQ) gratefully acknowledges the financial support of Queensland Health, Australia. This research was led by J.F.M. and supported by the ARC Laureate Fellowship (Grant FL200100028).

## Conflicts of Interest

The authors declare no conflicts of interest.

## Supporting information


**Table S1:** Common names, CAS numbers, absorption, metabolism, bioavailability, acceptable daily intake (ADI), sweetness equivalents to sucrose (SE) and urinary excretion rates (ER) of the sweeteners.
**Table S2:** Number of pools available for each age, sex & remoteness factor.
**Figure S1:** Concentrations of artificial sweeteners in urine pooled by sex & remoteness, regardless of age; number of pools for the different remoteness factors and sex were *n*_*female*(*InnerRegional*)_ = 15, *n*_*male*(*OuterRegional*)_ = 14, *n*_*female*(*OuterRegional*)_ = 14, *n*_*male*(*Remote*)_ = 7, *n*_*female*(*Remote*)_ = 13, *n*_*male*(*VeryRemote*)_ = 8, *n*_*female*(*VeryRemote*)_.
**Table S3:** The Mann Whitney test outcomes and descriptive statistics of the sex comparison.
**Table S4:** The Kruskal‐Wallis test and the post hoc pairwise comparisons outcomes using Dunn's test for age groups regardless of sex and remoteness.
**Table S5:** The Kruskal‐Wallis test and the post hoc pairwise comparisons outcomes using Dunn's test for age groups and sex, regardless of remoteness.
**Table S6:** The Kruskal‐Wallis test and the post hoc pairwise comparisons outcomes using Dunn's test for remoteness regardless of sex and age group.
**Table S7:** The Kruskal‐Wallis test and the post hoc pairwise comparisons outcomes using Dunn's test for remoteness and sex regardless of age group.
**Table S8:** Parameters and measured values per age group used for estimation of the yearly population intake.

## Data Availability

Data supporting the findings of this study are provided in the Supporting Information. More detailed datasets are available from the corresponding authors upon request.
