# Supplementary material for: Monitoring Population Exposure to Low‐ and No‐Calorie Sweeteners via Pooled Urine Analysis
Source: Food Sci Nutr. 2026 Apr 21;14(4):e71795. doi: 10.1002/fsn3.71795 (PMC13099274; doi:10.1002/fsn3.71795)
Supplement: Supplementary file 1 — Table S1: Common names, CAS numbers, absorption, metabolism, bioavailability, acceptable daily intake (ADI), sweetness equivalents to sucrose (SE) and urinary excretion rates (ER) of the sweeteners. Table S2: Number of pools available for each age, sex & remoteness factor. Figure S1: Concentrations of artificial sweeteners in urine pooled by sex & remoteness, regardless of age; number of pools for the different remoteness factors and sex were n female(InnerRegional) = 15, n male(OuterRegional) = 14, n female(OuterRegional) = 14, n male(Remote) = 7, n female(Remote) = 13, n male(VeryRemote) = 8, n female(VeryRemote). Table S3: The Mann Whitney test outcomes and descriptive statistics of the sex comparison. Table S4: The Kruskal‐Wallis test and the post hoc pairwise comparisons outcomes using Dunn's test for age groups regardless of sex and remoteness. Table S5: The Kruskal‐Wallis test and the post hoc pairwise comparisons outcomes using Dunn's test for age groups and sex, regardless of remoteness. Table S6: The Kruskal‐Wallis test and the post hoc pairwise comparisons outcomes using Dunn's test for remoteness regardless of sex and age group. Table S7: The Kruskal‐Wallis test and the post hoc pairwise comparisons outcomes using Dunn's test for remoteness and sex regardless of age group. Table S8: Parameters and measured values per age group used for estimation of the yearly population intake. [file FSN3-14-e71795-s001.docx]

**Monitoring Population Exposure to Low- and No-Calorie Sweeteners via Pooled Urine Analysis**

Nicole S. Schröter*a*, Jake W. O’Brien*a*, Richard Bade*a*, Leisa-Maree L. Toms*b*, Peter Hobson*c*, Daman Langguth*c*, Jochen F. Mueller*a,d*

*a*Queensland Alliance for Environmental Health Sciences (QAEHS), The University of Queensland, 20 Cornwall Street, Woolloongabba, Queensland 4102, Australia

*b*School of Public Health and Social Work, Queensland University of Technology, Kelvin Grove 4059, QLD, Australia

*c*Sullivan Nicolaides Pathology, 24 Hurworth Street, Bowen Hills, 4006, QLD, Australia

*d*Minderoo Centre - Plastics and Human Health, 20 Cornwall Street, Woolloongabba 4102, QLD, Australia Corresponding Author: [n.schroeter@uq.edu.au](mailto:n.schroeter@uq.edu.au)

# Supplement Material

## Standards

Table S1: Common names, CAS numbers, absorption, metabolism, bioavailability, acceptable daily intake (ADI), sweetness equivalents to sucrose (SE) and urinary excretion rates (ER) of the sweeteners; The ADI in milligrams per kilogram body weight per day according to the FDA and JEFCA; ER values used for calculations and the range found in literature, expressed as a percentage of the ingested dose considering bioavailability.

| Sweetener | CAS Number | Absorption | Metabolism | Bioavailability | ADI  [mg/kgbw/d] | SE to  Sucrose | ER value used  (range) [%] | References |
| --- | --- | --- | --- | --- | --- | --- | --- | --- |
| Acesulfame potassium | 55589-62-3 | Rapid, extensive | None | High (∼100%) | 15 | 200x | 99 (98-100) | (von Rymon Lipinski, 1985; Joint FAO/WHO Expert Committee on Food Additives, 1991; Volz et al., 1991; Cas-  tle et al., 2025) |
| Sodium  cyclamate | 139-05-9 | Partial | Bacterial conversion  to cyclohexylamine | Moderate (30-50%) | 11 | 40x | 40 (30-50) | (Renwick & Williams, 1972) |
| Saccharin | 81-07-2 | Rapid, extensive | None | High (80-100%) | 5 | 300x | 92 (85-97) | (McChesney & Golberg, 1973; Byard et al., 1974; Ball et al., 1977; Renwick,  1985) |
| Sucralose | 56038-13-2 | Minimal | None | Low (<15%) | 5 | 600x | 13 (8.9-22) | (Roberts et al., 2000; Grice & Gold-  smith, 2000; McLean Baird et al., 2000) |

**Samples**

Table S2: Number of pools available for each age, sex & remoteness factor. Each pool contains 25 individuals.

| Remoteness | Age Group | No. Pools | | Remoteness | Age Group | No. Pools | |
| --- | --- | --- | --- | --- | --- | --- | --- |
|  |  | Female | Male |  |  | Female | Male |
|  | 0 - <5 | 2 | 2 |  | 0 - <5 | 1 | 0 |
| Major Cities | 5 - <15  15 - <30  30 - <45 | 2  2  6 | 2  2  2 | Remote | 5 - <15  15 - <30  30 - <45 | 1  2  3 | 0  1  1 |
|  | 45 - <60 | 2 | 2 |  | 45 - <60 | 2 | 2 |
|  | >60 | 4 | 5 |  | >60 | 4 | 3 |
|  | 0 - <5 | 2 | 2 |  | 0 - <5 | 0 | 0 |
| Inner Regional | 5 - <15  15 - <30  30 - <45 | 2  2  2 | 2  2  2 | Very Remote | 5 - <15  15 - <30  30 - <45 | 1  5  3 | 0  2  2 |
|  | 45 - <60 | 2 | 2 |  | 45 - <60 | 3 | 2 |
|  | >60 | 5 | 6 |  | >60 | 3 | 2 |
|  | 0 - <5 | 2 | 2 |  |  |  |  |
|  | 5 - <15 | 2 | 2 |  |  |  |  |
| Outer Regional | 15 - <30  30 - <45 | 2  2 | 2  2 |  |  |  |  |
|  | 45 - <60 | 2 | 2 |  |  |  |  |
|  | >60 | 4 | 4 |  |  |  |  |

**Results**

### Remoteness

**
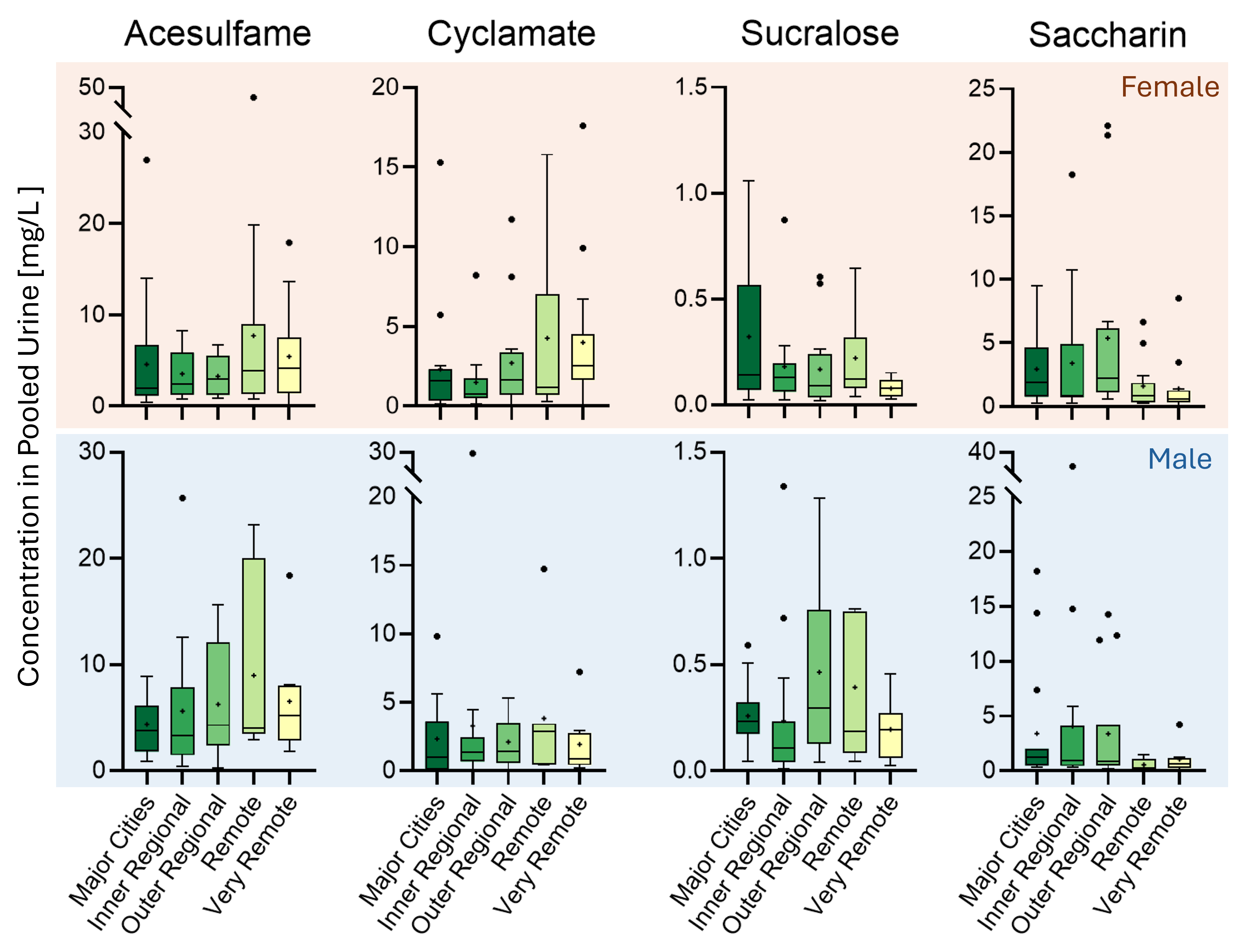
**

Figure S1: Concentrations of artificial sweeteners in urine pooled by sex & remoteness, regardless of age; number of pools for the different remoteness factors and sex were *n_male_*_(_*_MajorCities_*_)_ = 15, *nfemale*(*MajorCities*) = 18, *nmale*(*InnerRegional*) = 16, *nfemale*(*InnerRegional*) = 15, *nmale*(*OuterRegional*) = 14, *nfemale*(*OuterRegional*) = 14, *nmale*(*Remote*) = 7, *nfemale*(*Remote*) = 13, *nmale*(*VeryRemote*) = 8, *n_female_*_(_*_VeryRemote_*_)_ = 15 for acesulfame, cyclamate, sucralose and saccharin, with the following exceptions for cyclamate *n_male_*_(_*_OuterRegional_*_)_ = 13, *n_female_*_(_*_Remote_*_)_ = 12, and for sucralose *n_female_*_(_*_InnerRegional_*_)_ = 14, *n_female_*_(_*_Remote_*_)_ = 12; each pool consisted of 25 individuals. Data are presented as mean (+) and median (line) with ranges (whiskers) displayed in a box plot and outliers represented as dots, calculated using the ROUT method (Q=1%); female = upper row (red background), male = bottom row (blue background).

**Statistical Analysis**

Statistical significance for all test was denoted as follows: *p <* 0*.*001 (***), 0*.*001 *≤ p <* 0*.*01 (**), and 0*.*01 *≤ p <* 0*.*05 (*) with *p <* 0*.*0001 (****) indicating the strongest significance and ns = non-significant was donated *p ≥* 0*.*05.

### Sex

Table S3: The Mann Whitney test outcomes and descriptive statistics with mean, median, and adjusted Fisher-Pearson coefficient of Skewness (G1) of the sex comparison regardless of remoteness and age group.

| Results | Acesulfame | Saccharin | Sucralose | Cyclamate |
| --- | --- | --- | --- | --- |
| p-value | 0.0278 | 0.0949 | 0.0064 | 0.8031 |
| Significance | * | ns | ** | ns |
| Median of Female | 2.864 (n=75) | 1.224 (n=75) | 0.102 (n=73) | 1.612 (n=74) |
| Mean of Female | 4.836 (n=75) | 2.942 (n=75) | 0.198 (n=73) | 2.872 (n=74) |
| G1 Female | 3.4 (n=75) | 2.9 (n=75) | 2.1 (n=73) | 2.3 (n=74) |
| Median of Male | 3.813 (n=60) | 0.858 (n=60) | 0.217 (n=60) | 1.428 (n=59) |
| Mean of Male | 5.980 (n=60) | 2.933 (n=60) | 0.308 (n=60) | 2.666 (n=59) |
| G1 Male | 1.8 (n=60) | 3.0 (n=60) | 1.7 (n=60) | 4.5 (n=59) |

### Age

Table S4: The Kruskal-Wallis test and the post hoc pairwise comparisons outcomes using Dunn’s test for age groups regardless of sex and remoteness; Kruskal-Wallis H-value *>* 11*.*07 indicates significance (*χ*^2^ = 11*.*07*, df* = 5*, α* = 0*.*05).

| Kruskal-Wallis test results | Acesulfame | | Saccharin | | Sucralose | | Cyclamate | |
| --- | --- | --- | --- | --- | --- | --- | --- | --- |
| p-value | 0.0647 | | <0.0001 | | 0.0095 | | 0.0185 | |
| Significance | ns | | **** | | ** | | * | |
| H-value | 10.40 | | 62.06 | | 15.21 | | 13.58 | |
| Dunn’s Pairs Age Groups | Significance | p-value | Significance | p-value | Significance | p-value | Significance | p-value |
| 0-<5 vs. 5-<15 | ns | >0.9999 | ns | >0.9999 | ns | >0.9999 | ns | >0.9999 |
| 0-<5 vs. 15-<30 | ns | >0.9999 | **** | <0.0001 | ns | >0.9999 | ns | >0.9999 |
| 0-<5 vs. 30-<45 | ns | >0.9999 | **** | <0.0001 | ns | 0.3688 | ns | >0.9999 |
| 0-<5 vs. 45-<60 | ns | 0.6507 | **** | <0.0001 | ns | 0.2855 | ns | >0.9999 |
| 0-<5 vs. >60 | ns | >0.9999 | **** | <0.0001 | * | 0.0422 | ns | >0.9999 |
| 5-<15 vs. 15-<30 | ns | >0.9999 | **** | <0.0001 | ns | >0.9999 | ns | >0.9999 |
| 5-<15 vs. 30-<45 | ns | >0.9999 | **** | <0.0001 | ns | 0.6821 | ns | >0.9999 |
| 5-<15 vs. 45-<60 | ns | 0.5776 | ** | 0.0051 | ns | 0.5284 | ns | >0.9999 |

Table S4: The Kruskal-Wallis test and the post hoc pairwise comparisons outcomes using Dunn’s test for age groups regardless of sex and remoteness; Kruskal-Wallis H-value *>* 11*.*07 indicates significance (*χ*^2^ = 11*.*07*, df* = 5*, α* = 0*.*05).

| Kruskal-Wallis test results | Acesulfame | | Saccharin | | Sucralose | | Cyclamate | |
| --- | --- | --- | --- | --- | --- | --- | --- | --- |
| 5-<15 vs. >60 | ns | >0.9999 | ** | 0.0027 | ns | 0.0902 | ns | >0.9999 |
| 15-<30 vs. 30-<45 | ns | >0.9999 | ns | >0.9999 | ns | >0.9999 | ns | >0.9999 |
| 15-<30 vs. 45-<60 | ns | >0.9999 | ns | >0.9999 | ns | >0.9999 | ns | >0.9999 |
| 15-<30 vs. >60 | ns | >0.9999 | ns | >0.9999 | ns | 0.8700 | ns | >0.9999 |
| 30-<45 vs. 45-<60 | ns | 0.2188 | ns | >0.9999 | ns | >0.9999 | ns | 0.6229 |
| 30-<45 vs. >60 | ns | 0.5442 | ns | 0.4498 | ns | >0.9999 | ** | 0.0036 |
| 45-<60 vs. >60 | ns | >0.9999 | ns | >0.9999 | ns | >0.9999 | ns | >0.9999 |

Table S5: The Kruskal-Wallis test and the post hoc pairwise comparisons outcomes using Dunn’s test for age groups and sex, regardless of remoteness; Kruskal-Wallis H-value *>* 11*.*07 indicates significance (*χ*^2^ = 11*.*07*, df* = 5*, α* = 0*.*05).

| Kruskal-Wallis test results | Acesulfame | | Saccharin | | Sucralose | | Cyclamate | |
| --- | --- | --- | --- | --- | --- | --- | --- | --- |
|  | Female | Male | Female | Male | Female | Male | Female | Male |
| p-value | 0.2191 | 0.2843 | <0.0001 | <0.0001 | 0.0449 | 0.1538 | 0.2675 | 0.0539 |
| Significant | ns | ns | **** | **** | * | ns | ns | ns |
| H-value | 7.021 | 6.232 | 36.05 | 28.2 | 11.35 | 8.044 | 6.42 | 10.87 |
| Dunn’s Pairs Female | Significance | p-value | Significance | p-value | Significance | p-value | Significance | p-value |
| 0-<5 vs. 5-<15 | ns | >0.9999 | ns | >0.9999 | ns | >0.9999 | ns | >0.9999 |
| 0-<5 vs. 15-<30 | ns | >0.9999 | ** | 0.0011 | ns | >0.9999 | ns | >0.9999 |
| 0-<5 vs. 30-<45 | ns | >0.9999 | **** | <0.0001 | ns | 0.2703 | ns | >0.9999 |
| 0-<5 vs. 45-<60 | ns | >0.9999 | * | 0.0228 | ns | 0.4128 | ns | >0.9999 |
| 0-<5 vs. >60 | ns | >0.9999 | ** | 0.0044 | ns | 0.1976 | ns | >0.9999 |
| 5-<15 vs. 15-<30 | ns | >0.9999 | ** | 0.0047 | ns | >0.9999 | ns | >0.9999 |
| 5-<15 vs. 30-<45 | ns | >0.9999 | *** | 0.0002 | ns | 0.5332 | ns | >0.9999 |
| 5-<15 vs. 45-<60 | ns | >0.9999 | ns | 0.0776 | ns | 0.7754 | ns | >0.9999 |
| 5-<15 vs. >60 | ns | >0.9999 | * | 0.0186 | ns | 0.3979 | ns | >0.9999 |
| 15-<30 vs. 30-<45 | ns | >0.9999 | ns | >0.9999 | ns | >0.9999 | ns | 0.5675 |
| 15-<30 vs. 45-<60 | ns | >0.9999 | ns | >0.9999 | ns | >0.9999 | ns | >0.9999 |
| 15-<30 vs. >60 | ns | >0.9999 | ns | >0.9999 | ns | >0.9999 | ns | >0.9999 |
| 30-<45 vs. 45-<60 | ns | 0.2713 | ns | >0.9999 | ns | >0.9999 | ns | >0.9999 |
| 30-<45 vs. >60 | ns | >0.9999 | ns | >0.9999 | ns | >0.9999 | ns | 0.4696 |
| 45-<60 vs. >60 | ns | >0.9999 | ns | >0.9999 | ns | >0.9999 | ns | >0.9999 |
| Dunn’s Pairs Male | Significance | p-value | Significance | p-value | Significance | p-value | Significance | p-value |
| 0-<5 vs. 5-<15 | ns | >0.9999 | ns | >0.9999 | ns | >0.9999 | ns | >0.9999 |
| 0-<5 vs. 15-<30 | ns | >0.9999 | *** | 0.0004 | ns | >0.9999 | ns | >0.9999 |
| 0-<5 vs. 30-<45 | ns | >0.9999 | *** | 0.0005 | ns | >0.9999 | ns | >0.9999 |

Table S5: The Kruskal-Wallis test and the post hoc pairwise comparisons outcomes using Dunn’s test for age groups and sex, regardless of remoteness; Kruskal-Wallis H-value *>* 11*.*07 indicates significance (*χ*^2^ = 11*.*07*, df* = 5*, α* = 0*.*05).

| 0-<5 vs. 45-<60 | Acesulfame | | Saccharin | | Sucralose | | Cyclamate | |
| --- | --- | --- | --- | --- | --- | --- | --- | --- |
|  | ns | 0.6713 | * | 0.0111 | ns | >0.9999 | ns | >0.9999 |
| 0-<5 vs. >60 | ns | 0.4629 | * | 0.0106 | ns | >0.9999 | ns | 0.9004 |
| 5-<15 vs. 15-<30 | ns | >0.9999 | * | 0.0282 | ns | >0.9999 | ns | >0.9999 |
| 5-<15 vs. 30-<45 | ns | >0.9999 | * | 0.0332 | ns | >0.9999 | ns | >0.9999 |
| 5-<15 vs. 45-<60 | ns | >0.9999 | ns | 0.3694 | ns | >0.9999 | ns | >0.9999 |
| 5-<15 vs. >60 | ns | >0.9999 | ns | 0.4898 | ns | >0.9999 | ns | >0.9999 |
| 15-<30 vs. 30-<45 | ns | >0.9999 | ns | >0.9999 | ns | >0.9999 | ns | >0.9999 |
| 15-<30 vs. 45-<60 | ns | >0.9999 | ns | >0.9999 | ns | >0.9999 | ns | >0.9999 |
| 15-<30 vs. >60 | ns | >0.9999 | ns | >0.9999 | ns | 0.3198 | ns | 0.3184 |
| 30-<45 vs. 45-<60 | ns | >0.9999 | ns | >0.9999 | ns | >0.9999 | ns | >0.9999 |
| 30-<45 vs. >60 | ns | >0.9999 | ns | >0.9999 | ns | >0.9999 | ns | 0.1019 |
| 45-<60 vs. >60 | ns | >0.9999 | ns | >0.9999 | ns | >0.9999 | ns | >0.9999 |

### Remoteness

Table S6: The Kruskal-Wallis test and the post hoc pairwise comparisons outcomes using Dunn’s test for remoteness regardless of sex and age group; Kruskal-Wallis H-value *>* 9*.*488 indicates significance (*χ*^2^ = 9*.*488*, df* = 4*, α* = 0*.*05).

| Kruskal-Wallis test results | Acesulfame | | Saccharin | | Sucralose | | Cyclamate | |
| --- | --- | --- | --- | --- | --- | --- | --- | --- |
| p-value | 0.5791 | | 0.0062 | | 0.0248 | | 0.4204 | |
| Significance | ns | | ** | | * | | ns | |
| H-value | 2.874 | | 14.39 | | 11.17 | | 3.895 | |
| Dunn’s Pairs Remoteness | Significance | p-value | Significance | p-value | Significance | p-value | Significance | p-value |
| Major Cities vs. Inner Regional | ns | >0.9999 | ns | >0.9999 | ns | 0.3868 | ns | >0.9999 |
| Major Cities vs. Outer Regional | ns | >0.9999 | ns | >0.9999 | ns | >0.9999 | ns | >0.9999 |
| Major Cities vs. Remote | ns | >0.9999 | ns | 0.1533 | ns | >0.9999 | ns | >0.9999 |
| Major Cities vs. Very Remote | ns | >0.9999 | ns | 0.136 | * | 0.0378 | ns | >0.9999 |
| Inner Regional vs. Outer Regional | ns | >0.9999 | ns | >0.9999 | ns | >0.9999 | ns | >0.9999 |
| Inner Regional vs. Remote | ns | >0.9999 | ns | 0.414 | ns | >0.9999 | ns | >0.9999 |
| Inner Regional vs. Very Remote | ns | >0.9999 | ns | 0.3896 | ns | >0.9999 | ns | >0.9999 |
| Outer Regional vs. Remote | ns | >0.9999 | ns | 0.0553 | ns | >0.9999 | ns | >0.9999 |
| Outer Regional vs. Very Remote | ns | >0.9999 | * | 0.047 | ns | 0.2753 | ns | >0.9999 |
| Remote vs. Very Remote | ns | >0.9999 | ns | >0.9999 | ns | 0.2628 | ns | >0.9999 |

Table S7: The Kruskal-Wallis test and the post hoc pairwise comparisons outcomes using Dunn’s test for remoteness and sex regardless of age group; Kruskal-Wallis H-value *>* 9*.*488 indicates significance (*χ*^2^ = 9*.*488*, df* = 4*, α* = 0*.*05).

| Kruskal-Wallis test results | Acesulfame | | Saccharin | | Sucralose | | Cyclamate | |
| --- | --- | --- | --- | --- | --- | --- | --- | --- |
|  | Female | Male | Female | Male | Female | Male | Female | Male |
| p-value | 0.7619 | 0.6744 | 0.0184 | 0.1155 | 0.0596 | 0.1308 | 0.2712 | 0.8708 |
| Significance | ns | ns | * | ns | ns | ns | ns | ns |
| H-value | 1.858 | 2.335 | 11.86 | 7.416 | 9.061 | 7.099 | 5.161 | 1.244 |
| Dunn’s Pairs Female | Significance | p-value | Significance | p-value | Significance | p-value | Significance | p-value |
| Major Cities vs. Inner Regional | ns | >0.9999 | ns | >0.9999 | ns | >0.9999 | ns | >0.9999 |
| Major Cities vs. Outer Regional | ns | >0.9999 | ns | >0.9999 | ns | 0.8943 | ns | >0.9999 |
| Major Cities vs. Remote | ns | >0.9999 | ns | >0.9999 | ns | >0.9999 | ns | >0.9999 |
| Major Cities vs. Very Remote | ns | >0.9999 | ns | 0.2188 | ns | 0.0623 | ns | >0.9999 |
| Inner Regional vs. Outer Regional | ns | >0.9999 | ns | >0.9999 | ns | >0.9999 | ns | >0.9999 |
| Inner Regional vs. Remote | ns | >0.9999 | ns | >0.9999 | ns | >0.9999 | ns | >0.9999 |
| Inner Regional vs. Very Remote | ns | >0.9999 | ns | >0.9999 | ns | >0.9999 | ns | 0.3008 |
| Outer Regional vs. Remote | ns | >0.9999 | ns | 0.1741 | ns | >0.9999 | ns | >0.9999 |
| Outer Regional vs. Very Remote | ns | >0.9999 | * | 0.0235 | ns | >0.9999 | ns | >0.9999 |
| Remote vs. Very Remote | ns | >0.9999 | ns | >0.9999 | ns | 0.3093 | ns | >0.9999 |
| Dunn’s Pairs Male | Significance | p-value | Significance | p-value | Significance | p-value | Significance | p-value |
| Major Cities vs. Inner Regional | ns | >0.9999 | ns | >0.9999 | ns | >0.9999 | ns | >0.9999 |
| Major Cities vs. Outer Regional | ns | >0.9999 | ns | >0.9999 | ns | >0.9999 | ns | >0.9999 |
| Major Cities vs. Remote | ns | >0.9999 | ns | 0.2389 | ns | >0.9999 | ns | >0.9999 |
| Major Cities vs. Very Remote | ns | >0.9999 | ns | >0.9999 | ns | >0.9999 | ns | >0.9999 |
| Inner Regional vs. Outer Regional | ns | >0.9999 | ns | >0.9999 | ns | 0.1362 | ns | >0.9999 |
| Inner Regional vs. Remote | ns | >0.9999 | ns | 0.2124 | ns | >0.9999 | ns | >0.9999 |
| Inner Regional vs. Very Remote | ns | >0.9999 | ns | >0.9999 | ns | >0.9999 | ns | >0.9999 |
| Outer Regional vs. Remote | ns | >0.9999 | ns | 0.3953 | ns | >0.9999 | ns | >0.9999 |
| Outer Regional vs. Very Remote | ns | >0.9999 | ns | >0.9999 | ns | >0.9999 | ns | >0.9999 |
| Remote vs. Very Remote | ns | >0.9999 | ns | >0.9999 | ns | >0.9999 | ns | >0.9999 |

## Estimation

Table S8: Parameters and measured values per age group used for estimation of the yearly population intake (Intake*_population_*) including the proportional weight of each segment relative to the overall population of 25,510,998 (Australian Bureau of Statistics (ABS), 2019); V*urine* = urinary volume according to the age group (Beckford et al., 2019; González-Mariño et al., 2017); EF = excretion factor is the fraction of what was excreted in urine after 24 h of ingestion; c*_measured_* = measured mean excreted concentration (excluding outliers) in the urine pools; c*_intake_* = calculated intake concentration considering the excretion rate for each compound; Intake*_daily_* = calculated daily intake per capita considering the urinary volume; Intake*_yearly_* = calculated yearly intake per capita.

| Compound | Age  Group  years | V*urine*  L/d | EF | c*measured*  mg/L | c*intake*  mg/L | Intake*_daily_*  per capita  mg/d/person | Intake*yearly*  per capita  g/year/person | Population | Intake*population* per age group  kg/year | Part of the  Whole  % |
| --- | --- | --- | --- | --- | --- | --- | --- | --- | --- | --- |
| Saccharin | 0-<5 | 0.53 | 0.92 | 14.8 | 16.1 | 8.53 | 3.11 | 1547269 | 4818 | 16 |
|  | 5-<15 | 1.57 | 0.92 | 5.66 | 6.16 | 9.42 | 3.44 | 3201327 | 11005 | 36 |
|  | 15-<30 | 1.57 | 0.92 | 1.21 | 1.32 | 2.06 | 0.75 | 5104173 | 3847 | 13 |
|  | 30-<45 | 1.57 | 0.92 | 0.86 | 0.93 | 1.47 | 0.54 | 5327636 | 2853 | 9 |
|  | 45-<60 | 1.57 | 0.92 | 1.24 | 1.35 | 2.11 | 0.77 | 4802401 | 3706 | 12 |
|  | >60 | 1.57 | 0.92 | 1.27 | 1.38 | 2.17 | 0.79 | 5528192 | 4377 | 14 |
| Acesulfame | 0-<5 | 0.53 | 0.99 | 3.39 | 3.42 | 1.81 | 0.66 | 1547269 | 1025 | 1 |
|  | 5-<15 | 1.57 | 0.99 | 3.63 | 3.66 | 5.75 | 2.10 | 3201327 | 6717 | 9 |
|  | 15-<30 | 1.57 | 0.99 | 5.51 | 5.57 | 8.74 | 3.19 | 5104173 | 16288 | 21 |
|  | 30-<45 | 1.57 | 0.99 | 5.41 | 5.47 | 8.58 | 3.13 | 5327636 | 16690 | 21 |
|  | 45-<60 | 1.57 | 0.99 | 6.81 | 6.88 | 10.8 | 3.94 | 4802401 | 18933 | 24 |
|  | >60 | 1.57 | 0.99 | 5.68 | 5.73 | 9.00 | 3.29 | 5528192 | 18163 | 23 |
| Cyclamate | 0-<5 | 0.53 | 0.40 | 2.46 | 6.15 | 3.26 | 1.19 | 1547269 | 2537 | 2 |
|  | 5-<15 | 1.57 | 0.40 | 2.73 | 6.82 | 10.7 | 3.91 | 3201327 | 16625 | 13 |
|  | 15-<30 | 1.57 | 0.40 | 2.52 | 6.29 | 9.88 | 3.61 | 5104173 | 40313 | 19 |
|  | 30-<45 | 1.57 | 0.40 | 1.95 | 4.88 | 7.66 | 2.80 | 5327636 | 41307 | 15 |
|  | 45-<60 | 1.57 | 0.40 | 3.63 | 9.08 | 14.3 | 5.20 | 4802401 | 46860 | 26 |
|  | >60 | 1.57 | 0.40 | 3.13 | 7.83 | 12.3 | 4.49 | 5528192 | 44953 | 25 |
| Sucralose | 0-<5 | 0.53 | 0.13 | 0.40 | 3.06 | 1.62 | 0.59 | 1547269 | 915 | 3 |
|  | 5-<15 | 1.57 | 0.13 | 0.31 | 2.40 | 3.77 | 1.38 | 3201327 | 4407 | 17 |
|  | 15-<30 | 1.57 | 0.13 | 0.32 | 2.47 | 3.89 | 1.42 | 5104173 | 7238 | 28 |
|  | 30-<45 | 1.57 | 0.13 | 0.23 | 1.78 | 2.79 | 1.02 | 5327636 | 5420 | 21 |
|  | 45-<60 | 1.57 | 0.13 | 0.20 | 1.54 | 2.42 | 0.88 | 4802401 | 4236 | 16 |
|  | >60 | 1.57 | 0.13 | 0.17 | 1.28 | 2.01 | 0.73 | 5528192 | 4062 | 15 |

# References

von Rymon Lipinski, G. W. (1985). The new intense sweetener Acesulfame K. *Food Chemistry*, *16* (3-4), 259–269. <https://doi.org/10.1016/0308-8146(85)90120-7> Joint FAO/WHO Expert Committee on Food Additives. (1991). Toxicological evaluation of certain food additives and contaminants. *The 37th Meeting*, *28*, 183–218. Volz, M., Christ, O., Eckert, H., Herok, J., Kellner, M., & Rupp, W. (1991). Kinetics and Biotransformation of Acesulfame-K. In *Acesulfame-k* (pp. 7–26). Marcel

Dekker, Inc.

Castle, L., Andreassen, M., Aquilina, G., Bastos, M. L., Boon, P., Fallico, B., FitzGerald, R., Frutos Fernandez, M. J., Grasl-Kraupp, B., Gundert-Remy, U., Gürtler, R., Houdeau, E., Kurek, M., Louro, H., Morales, P., Passamonti, S., Batke, M., Bruzell, E., Chipman, J., . . . Lodi, F. (2025). Re-evaluation of acesulfame K (E 950) as food additive. *EFSA Journal*, *23* (4). <https://doi.org/10.2903/j.efsa.2025.9317>

Renwick, A. G., & Williams, R. T. (1972). The Fate of Cyclamate in Man and Other Species. *Biochem. J*, *129*, 869–879. <http://portlandpress.com/biochemj/article->pdf/129/4/869/778964/bj1290869.pdf

McChesney, E. W., & Golberg, L. (1973). The Excretion and Metabolism of Saccharin in Man. I. Methods of investigation and Preliminary Results*

. *Food and Cosmetics Toxicology*, *11*, 403–414.

Byard, J. L., McChesney, E. W., Golberg, L., & Coulston, F. (1974). Excretion and metabolism of saccharin in man. II. Studies with 14C-labelled and unlabelled saccharin. *Food and Cosmetics Toxicology*, *12* (2), 175–184. <https://doi.org/10.1016/0015-6264(74)90362-9>

Ball, L. M., Renwick, A. G., & Williams, R. T. (1977). The Fate of [14C]Saccharin in Man, Rat and Rabbit and of 2-Sulphamoyl[14C]benzoic Acid in the Rat.

*Xenobiotica*, *7* (4), 189–203. <https://doi.org/10.3109/00498257709035778>

Renwick, A. G. (1985). The disposition of saccharin in animals and man—A review. *Food and Chemical Toxicology*, *23* (4-5), 429–435. <https://doi.org/10.1016/0278->6915(85)90136-X

Roberts, A., Renwick, A. G., Sims, J., & Snodin, D. J. (2000). Sucralose metabolism and pharmacokinetics in man. *Food and Chemical Toxicology*, *38* (SUPPL.2), 31–41. <https://doi.org/10.1016/S0278-6915(00)00026-0>

Grice, H. C., & Goldsmith, L. A. (2000). Sucralose—an overview of the toxicity data. *Food and Chemical Toxicology*, *38* (SUPPL.2), 1–6. <https://doi.org/10.1016/S0278->6915(00)00023-5

McLean Baird, I., Shephard, N. W., Merritt, R. J., & Hildick-Smith, G. (2000). Repeated dose study of sucralose tolerance in human subjects. *Food and Chemical Toxicology*, *38* (SUPPL.2), 123–129. <https://doi.org/10.1016/S0278-6915(00)00035-1>

Australian Bureau of Statistics (ABS). (2019). ERP by LGA (ASGS 2019), Age and Sex, 2001 to 2019 - Data Explorer. [https://dataexplorer.abs.gov.au/?fs%5B0%5D=](https://dataexplorer.abs.gov.au/?fs%5B0%5D) People%2C0%7CPopulation%23POPULATION%23&pg=0&fc=People&bp=true&snb=83

Beckford, K., Grimes, C. A., Margerison, C., Riddell, L. J., Skeaff, S. A., West, M. L., & Nowson, C. A. (2019). A systematic review and meta-analysis of 24-h urinary output of children and adolescents: impact on the assessment of iodine status using urinary biomarkers. *European Journal of Nutrition*, *59* (7), 3113. <https://doi.org/10.1007/S00394-019-02151-W>

González-Mariño, I., Rodil, R., Barrio, I., Cela, R., & Quintana, J. B. (2017). Wastewater-Based Epidemiology as a New Tool for Estimating Population Exposure to Phthalate Plasticizers. *Environmental Science and Technology*, *51* (7), 3902–3910. <https://doi.org/10.1021/ACS.EST.6B05612/ASSET/IMAGES/LARGE/ES->2016-05612Z{\_}0002.JPEG
